# Nurse Alice Petitt's Articulation

**Published:** 1918-11-30

**Authors:** 


*To the Editor of* The Hospital.

### Nurse Alice Petitt's Articulation.

*To the Editor of* The Hospital.

Sir,—May I be permitted to pay a tribute to Alice Petitt, member of the College of Nursing, your correspondent of last week? She is the kind of person who ought to be on the Executive of the College of Nursing. She sees the point, and makes no secret of the fact that she disseminates her views. I have not the pleasure of this lady's acquaintance, but I recognise in her sentiments the first sign of life, and I admire her most of all for her postscript: "I am not ashamed of my name; you may publish it."

I am convinced that for the British nurse it is now or never. In the future we shall have a fifth-rate Nursing Service, or a first-rate. If every town and hamlet in England produces an "Alice Petitt, member of the College of Nursing," free, fearless, and untrammelled, there will be no necessity for Lord Knutsford's issuing of Charters of Liberty. Emancipation from bondage will come as a matter of course. Ierne.

